# Oncogene addiction and radiation oncology: effect of radiotherapy with photons and carbon ions in ALK-EML4 translocated NSCLC

**DOI:** 10.1186/s13014-017-0947-0

**Published:** 2018-01-05

**Authors:** Ying Dai, Quanxiang Wei, Christian Schwager, Janina Hanne, Cheng Zhou, Klaus Herfarth, Stefan Rieken, Kenneth E. Lipson, Jürgen Debus, Amir Abdollahi

**Affiliations:** 1 German Cancer Consortium (DKTK), Heidelberg, Germany; 2 Divisions of Molecular & Translational Radiation Oncology and Thoracic Radiation Oncology, Heidelberg Ion Therapy Center (HIT), Heidelberg Institute of Radiation Oncology (HIRO), University of Heidelberg Medical School and National Center for Tumor Diseases (NCT), German Cancer Research Center (DKFZ), Im Neuenheimer Feld 450, 69120 Heidelberg, Germany; 3 FibroGen, Inc., San Francisco, CA 94158 USA; 4 Department of Oncology, the 1st Affiliated Hospital of Anhui Medical University, Hefei, China

**Keywords:** Non-small-cell lung cancer, EML4-ALK-fusion, ALK inhibitors, Radiotherapy, Carbon ions

## Abstract

**Background:**

Patients with Echinoderm microtubule-associated protein-like 4 (EML4)-anaplastic lymphoma kinase (ALK) positive lung cancer are sensitive to ALK-kinase inhibitors. TAE684 is a potent second generation ALK inhibitor that overcomes Crizotinib resistance. Radiotherapy is an integral therapeutic component of locally advanced lung cancer. Therefore, we sought to investigate the effects of combined radiotherapy and ALK-inhibition via TAE684 in ALK-positive vs. wild type lung cancer cells.

**Methods:**

Human non-small cell lung cancer (NSCLC) cell lines harboring wild-type ALK (A549), EML4-ALK translocation (H3122) and murine Lewis Lung Cancer (LLC) cells were investigated. Cells were irradiated with 1–4 Gy X-Rays (320 keV) and carbon ions (Spread-out Bragg Peak, SOBP (245.4–257.0 MeV/u)) at Heidelberg Ion Therapy center. TAE684 was administered at the dose range 0–100 nM. Clonogenic survival, proliferation and apoptosis via caspase 3/7 expression level were assessed in all three cell lines using time-lapse live microscopy.

**Results:**

TAE684 inhibited the proliferation of H3122 cells in a dose-dependent manner with a half maximal inhibitory concentration (IC_50_) of ~ 8.2 nM. However, A549 and LLC cells were relatively resistant to TAE684 and IC_50_ was not reached at concentrations tested (up to 100 nM) in proliferation assay. The antiproliferative effect of TAE684 was augmented by radiotherapy in H3122 cells. TAE684 significantly sensitized H3122 cells to particle therapy with carbon ions (sensitizer enhancement ratio ~1.61, *p* < 0.05). Caspase 3/7 activity was evidently enhanced after combination therapy in H3122 cells.

**Conclusions:**

This is the first report demonstrating synergistic effects of combined TAE684 and radiotherapy in EML4-ALK positive lung cancer cells. In addition to conventional photon radiotherapy, ALK-inhibition also enhanced the effects of particle irradiation using carbon ions. Our data indicate beneficial effects of combined ALK-inhibition and radiotherapy in treatment of this distinct subpopulation of NSCLC that warrant further evaluation.

## Background

Lung cancer is the leading cause of cancer mortality worldwide, and NSCLC comprises about 80% of lung cancer cases. Most patients are diagnosed with non-resectable diseases and approximately 1/3 present with locally advanced diseases (stage III) i.e., the tumor may exceed the structures of the lung itself and/or have spread to ipsilateral mediastinal and/or subcarinal lymph nodes, but no clinical evidence for distant metastasis is found [1]. Radiochemotherapy is an integral component of the multimodal treatment for these locally advanced patients. Despite improvements in radiotherapy delivery, different chemotherapy combination and schemes, the median survival in this relatively heterogenous collective population is ~ 21 months with 3 years survival rates of ~ 30% [2–8].

The chromosomal rearrangement between ALK and EML4 was first reported by Soda et al. from a resected specimen of a male lung adenocarcinoma patient [9]. Between 3% to 7% of NSCL tumors harbor the EML4-ALK fusion [10, 11]. It is predominantly detected in adenocarcinomas of light smokers (< 10 packs per year) or non-smokers at a younger age, and is independent of epidermal growth factor receptor (EGFR) or KRAS mutations [12]. The EML4-ALK fusion protein leads to an aberrant activation of the ALK tyrosine kinase and its related downstream signaling [13]. A number of interconnected pathways are involved in ALK downstream signaling among which the MAP Kinase pathways including Ras- ERK and phosphoinositide 3-kinase (PI3K)-Akt are best characterized [14]. Activation of ALK-mediated signaling pathways plays a key role in tumorigenic transformation of cells by promoting cell growth and inhibiting apoptosis, irrespective of the originating organ [15, 16]. Soda et al. have shown that cells overexpressing EML4-ALK are able to generate subcutaneous or lung orthotopic tumors in a nude mouse model [9, 17]. Another chromosomal translocation between the nucleophosmin (NPM) gene on chromosome 5q35 and ALK gene on 2p23 is expressed in 60%–70% of anaplastic large cell lymphoma (ALCLs) [13, 18, 19].

Based on the discoveries of ALK as an important oncogene and the encoded fusion protein in development of different cancers, a search for small molecular ALK-tyrosine kinase inhibitors (TKIs) identified Crizotinib (PF-02341066) as the first in class compound receiving FDA-approval for treatment of ALK-positive advanced lung cancer in 2011 [20]. However, Crizotinib was originally identified in a screening program searching for a c-Met receptor tyrosine kinase inhibitor (RTKi). Accordingly, Crizotinib is not considered a specific ALK-inhibitor (with a half maximal inhibitory concentration, IC_50_: 24 nM) and more potently inhibits other kinases such as c-Met (IC_50_: 11 nM) [21] and ROS1 (IC _50_: 1.7 nM) [10]. Moreover, a gatekeeper mutation in the active kinase domain (L1196 M) renders ALK-positive lung cancer cells resistant to Crizotinib therapy [22]. In contrast, ALK-positive NSCLC cells harboring this gatekeeper mutation remain highly sensitive to second generation ALK-inhibitors such as TAE684 [22, 23].

TAE684 is a potent and selective ALK-inhibitor with a reported IC_50_ of ~ 3 nM in ALK positive cell lines [24]. It was first reported to block the growth of ALCL-derived and ALK-dependent cell lines with IC_50_ values between 2 and 10 nM [25]. The inhibitory effects were also observed in NSCLC cell lines with IC_50_ values between 15 and 50 nM [23]. TAE684 was shown to induce apoptosis and cell cycle arrest via rapid and sustained inhibition of NPM-ALK phosphorylation and its downstream effectors including ERK, Akt and STAT3 and/or STAT5b [25].

Currently, ALK-inhibitors are approved only for advanced NSCLC. The translation of ALK-inhibitors in locally advanced NSCLC patients with EML4-ALK fusion will largely depend on a better understanding of the effects elicited by this novel class of drugs in combination with radiotherapy. Therefore, we aimed to explore the interaction of radiotherapy and ALK-inhibition by TAE684 in tumor cell lines with and without ALK-fusion. This is, to our knowledge the first report on beneficial effects of this combination in ALK-positive tumors. It further supports the concept of targeting oncogene addiction combined with radiotherapy, preferably with carbon ions, investigated at the German Research Foundation (DFG) “clinical research group heavy ion therapy (KFO-214)” within the project TP5 “Heavy Ions in Lung Cancer”.

## Methods

### Cells and cell culture

**Fig. 1:**
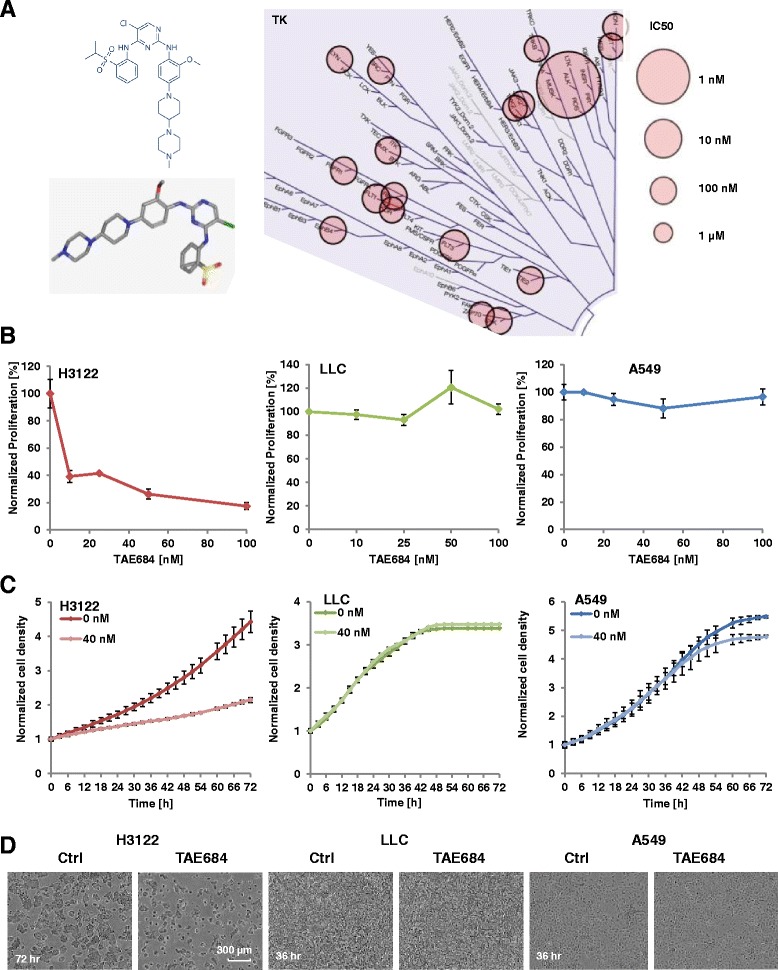
Selective antiproliferative effect of TAE684 in ALK-positive NSCLC. The kinase inhibition profile by TAE684 (IC_50_, mapped by TREE *spot*) as well as its 2D and 3D molecular structures (adapted from PubChem) are shown (**a**). Cell proliferation was assessed by counting viable cells 72 post treatment with a cell-permeant DNA-binding fluorescent dye (CyQuant-Direct) (**b**). Alternatively, cell proliferation was monitored longitudinally by live microscopy and the confluence levels as well as representative photomicrographs are presented (**c** and **d**). TAE684 potently inhibited cell proliferation in ALK-positive H3122 NSCLC but was less or not effective in A549 and LLC cells, respectively. Bars represent mean ± SD. TK: tyrosine kinase; Ctrl: control

LLC cells were purchased from ATCC, Manassas, USA; adenocarcinomic human alveolar basal epithelial (A549) cells were obtained from Deutsche Sammlung von Mikroorganismen und Zellkulturen GmbH (DSMZ) and the human NSCLC cell line H3122 was provided by Frederick National Laboratory for Cancer Research, Maryland, USA. A549 cells were cultured in Dulbecco’s modified Eagle’s Medium (DMEM) (Biochrom) containing 10% FBS as previously described [26]. LLC and H3122 cells were cultured in RPMI1640 medium supplemented with 10% fetal bovine serum (FBS) (Biochrom). For H3122, additional 1% L-glutamine (Sigma) was added. The ALK-inhibitor TAE684, C_30_H_40_ClN_7_O_3_S, with molecular weight 614.2017 [g/mol] was obtained from Absource Diagnostics GmbH (Selleckchem) and dissolved in ethanol. The 2D molecular structure and 3D conformer of TAE684 are adapted from PubChem (http://pubchem.ncbi.nlm.nih.gov, PubChem id: 16038120) and provided in Fig. 1a.

### Cell proliferation assay

H3122, LLC, and A549 cells were seeded in 96 well plates at 5000 cells/well. Cells were treated with TAE684 for 2 h before exposure to 4 Gy of irradiation. Cell confluence was monitored by time-lapse microscopy as previously described using IncuCyte ™ Zoom (Essen BioScience) with a 10X objective for 72 h [27]. In addition, fluorimetric measurement was performed using the CyQUANT^®^ Direct Cell Proliferation Assay (Life Technologies) according to manufacturers’ instruction (3 Fountain Drive Inchinnan Business Park, Paisley PA4 9RF, UK). In brief, cells proliferation was quantified 72 h post treatment using 2X detection reagent (11.7 mL PBS, 48 μL CyQUANT® Direct nucleic acid stain and 240 μL CyQUANT® Direct background suppressor I). Signal intensities after incorporation of fluorescent CyQuant dye were measured at 485/520 nm filter set by Infinite M200 Microplate reader (Tecan). The signal intensity from treated groups was normalized to the one of vehicle groups.

### Cell apoptosis assay

For time-lapse live microscopy of apoptosis, CellPlayer™ Kinetic Caspase-3/7 Assay (Essen BioScience) was used to measure the activity of the executer Caspase 3/7 as surrogate for apoptotic cell death. H3122 cells were seeded in 96 well plates at 5000 cells/ well. Cells were treated with 5 nM TAE684 for 2 h before exposure to 4 Gy of irradiation. DEVD-NucView™ 488 caspase-3 substrate (Biotium) was then added at a final concentration of 5 μM, and cells were monitored for 72 h with IncuCyte ™ Zoom using a 10X objective. At the end of the monitoring period, Vybrant DyeCycle Green (Life Technologies) diluted in PBS was added at a final concentration of 1 μM to determine the total number of cell nuclei (DNA-stain). Fluorescence was measured with excitation at 480 nm and detection at 544 nm. The average fluorescence signal per well was calculated as the total fluoresce intensity per well divided by the corresponding Vybrant DyeCycle Green signal intensity. The signal intensity from treated groups was normalized to the vehicle groups with or without irradiation, respectively.

### Clonogenic assay

A pilot experiment was performed to determine the plating efficacy for each cell type, i.e. detecting the number of colonies formed as the function of different cell densities. Based on these data, 75 to 10,000 cells depending on radiation dose was seeded in triplicate into T25 flasks containing 5 mL medium overnight and exposed to 4 nM TAE684or vehicle (final ethanol concentration of ≤0.05%) for 2 h. The cells were then irradiated with X-RAD 320 (Precision X-Ray; 320.0 keV/12.5 mA) at room temperature at a dose range of 0, 1, 2 and 4 Gy. Carbon irradiation was performed at the Heidelberg Ion Therapy Center (HIT) with the horizontal beamline using a raster scanning technique as described [28]. Cell monolayers were irradiated with 0, 1, 2, 4 Gy physical dose which were delivered as an extended SOPB of 10 mm at a water equivalent depth of 120 mm. Cells were subsequently cultured at 37 °C/ 5% CO_2_ until colonies consisting of at least 50 cells formed in the control culture (without TAE684 or irradiation). The colonies were fixed with 75% methanol and 25% acetic acid and stained with the 1 g/L crystal violet.

### Statistical data analysis

The kinase inhibition spectrum (IC_50_) of TAE684 was visualized (Fig. 1a) and mapped by TREE *spot*™ Compound Profile Visualization Tool (http://www.discoverx.com/services/drug-discovery-development-services/treespot-data-analysis) [25]. The number of colonies was quantified with Image J software (http://rsbweb.nih.gov/ij) and the survival fraction (SF) was estimated according to the formula: SF = number of colonies formed in test condition/(number of cells seeded × plating efficiency of control group). The sensitizer enhancement ratio (SER) was calculated as the surviving fraction in vehicle-treated cells divided by that for TAE684-treated cells. Clonogenic survival and SER were performed using the CS-Cal (www.oncoexpress.de). Student’s t-test was utilized to evaluate the significance between groups. *P* < 0.05 was significant. Data represent mean ± standard deviation (SD) if not otherwise indicated.

## Results

### Antiproliferative effects of TAE684 in NSCLC

To assess the effects of TAE684 on cell proliferation, EML4-ALK fusion positive H3122 cells as well as A549 and LLC (both EML4-ALK negative) were treated with TAE684 (0–100 nM) and relative proliferation was determined 72 h post treatment using the Cyquant assay. TAE684 potently inhibited H3122 cell proliferation with an IC_50_ ~ 8.2 nM (Fig. 1b). In contrast, A549 cells showed modest sensitivity with a U-shaped dose response and ~ 12% maximum inhibition at 40-50 nM (*p* < 0.05, Fig. 1b). LLC were resistant to TAE684 treatment in the dose range studied (up to 100 nM). In line with these observations, cell proliferation kinetic determined by time- lapse microscopy in 3 h intervals for a period of 72 h post therapy revealed a similar sensitivity pattern. A significant delay in H3122 cells was observed after 40 nM TAE684 treatment (*p* < 0.001, Fig. 1c and d). In contrast, 40 nM TAE684 does not affect LLC growth kinetic and elicited a moderate effect on A549 cells with a late onset (p < 0.001 at 72 h) and 13% reduction of cell confluence as compared to vehicle treated control (Fig. 1c and d). Together, these data confirm potent and selective antiproliferative effects of TAE684 in ALK positive NSCLC.

### TAE684 only augments antiproliferative effects of radiotherapy in ALK-positive NSCLC

**Fig. 2:**
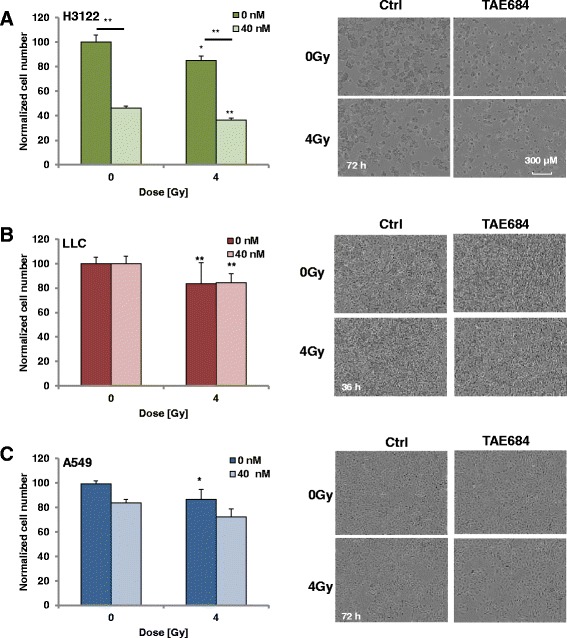
TAE684 selectively augments radiotherapy-induced antiproliferative effects in ALK positive NSCLC. Cell proliferation after incubation with vehicle or TAE684 (40 nM) alone or in combination with irradiation (4 Gy) was evaluated in H3122 (**a**), LLC (**b**) and A549 (**c**) cells (left panel). Only in ALK-positive H3122 cells dual treatment with TAE684 and radiotherapy reduced cell proliferation by 56% as compared to radiotherapy alone (*p* < 0.01). Photomircographs of representative fields after 72 h (H3122 and A549) or 36 h (LLC) are shown on the right panel. Cell numbers were normalized to that of non-treated groups for each cell line. Bars indicate mean ± SD. * *p* < 0.05 and ** *p* < 0.01

To evaluate the effects of dual treatment with TAE684 and radiation on cell proliferation, H3122, A549 and LLC cells were treated with TAE684 (40 nM) for 2 h prior to irradiation (4 Gy). Cell confluence was measured 72 h post treatment by microscopy. Radiotherapy exhibited a modest but significant inhibition of cell proliferation in H3122 (15% inhibition, *p* < 0.05, Fig. 2a), LLC cells (16% inhibition, *p* < 0.01, Fig. 2b) and A549 cells (13% inhibition, p < 0.05, Fig. 2c). Combined treatment with TAE684 and radiotherapy significantly reduced cell proliferation as compared to radiotherapy alone (56%, *p* < 0.01, Fig. 2a) but only in ALK-positive H3122 cells. In contrast, the same response was achieved by dual therapy in LLC cells (Fig. 2b) while the dual combination was less efficient in inhibiting cell proliferation in A549 cells as compared to radiotherapy alone (Fig. 2c). Representative photomicrographs showing the NSCLC cell confluence levels at 72 h (H3122 and A549) or 36 h (LLC) post therapy are presented.

### Synergistic effects of combined TAE684 & radiotherapy on NSCLC survival

**Fig. 3:**
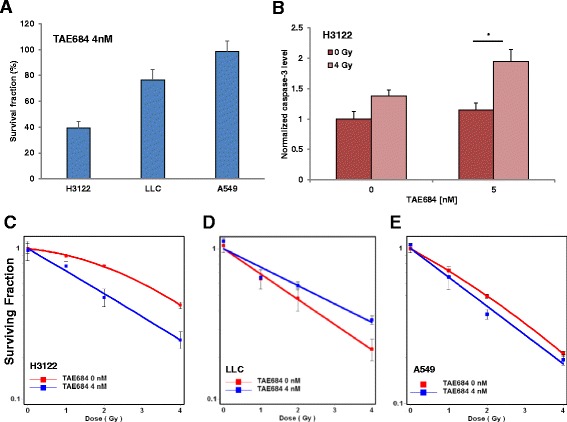
Synergistic effects of combined TAE684 and radiotherapy on NSCLC survival and apoptotic activity. The survival fraction after 4 nM TAE684 monotherapy revealed high sensitivity of H3122 cells and moderate response of LLC cells instead of A549 cells (**a**). In parallel, TAE684 induced apoptosis in H3122 cells and the addition of radiotherapy enhanced Caspase 3/7 level in a synergistic manner (**b**). H3122 (**c**), LLC (**d**) and A549 (**e**) cells were treated with vehicle or TAE684 (4 nM) and irradiated with 0, 1, 2 or 4 Gy. TAE684 selectively sensitized ALK-positive H3122 to radiotherapy. In contrast, a trend toward antagonistic effects was found in LLC cells treated with this dual combination. Bar represent mean ± SD

To further evaluate the effects of mono- and dual therapies, the clonogenic survival of all three NSCLC lines was assessed. The SF of ALK-positive H3122 cells was prominently reduced with SF~61% at 4 nM TAE684. Interestingly, a SF ~23% was achieved at 4 nM TAE684 in LLC cells that were otherwise found to be resistant in proliferation assay. At this dose level, the clonogenic survival of A549 cells was not affected (Fig. 3a). Comparative analysis across all three cell lines was performed using 4 nM TAE684 and a dose series of radiotherapy (0–4 Gy). The most significant radiosensitizing effect of TAE684 was found in the ALK-positive H3122 cells (Fig. 3c) with SER: 1.6 (*p* < 0.01) at SF50%. In LLC cells (Fig. 3d), TAE648 exerted a radioprotective effect. The protection enhancement ratio (PER) was 1.5 (p < 0.01) at SF 50% for combined treatment with TAE684 and irradiation vs. irradiation alone. TAE684 moderately sensitized A549 cells to irradiation with SER: 1.23 (*p* = 0.03, at SF50%) (Fig. 3e).

### TAE684 enhanced radiation induce apoptosis in H3122 cells

In order to understand the basis of reduced cell viability in the presence of TAE684 and/or irradiation, caspases 3/7 was examined as a marker of apoptosis. TAE684 alone induced 14% apoptosis compared to vehicle control in ALK-positive H3122 cells (Fig. 3b). Likewise, in irradiated groups, TAE684 enhanced caspase-3 activity by 41% as compared to the vehicle groups. Addition of 4Gy radiotherapy led to ~ 3-fold enhancement compared to TAE684 monotherapy in apoptotic activity (*p* < 0.05). Together, these data support potent radiosensitizing effects of TAE684 in ALK-positive H3122 NSCLC.

### TAE684 sensitizes ALK-positive NSCLC to carbon ions

**Fig. 4:**
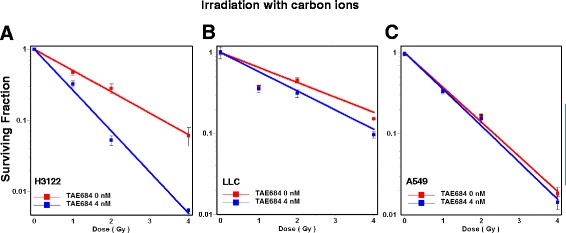
TAE684 sensitizes ALK positive NSCLC cells to carbon ions. The surviving fraction of H3122(**a**), LLC (**b**) and A549 (**c**) cells was determined after vehicle or TAE684 (4 nM) treatment and carbon ion irradiation (0, 1, 2 or 4 Gy). TAE684 treatment potently sensitized ALK-positive H3122 to carbon ion radiotherapy, while a moderate radiosensitivity was induced in LLC cells and no response was observed in A549 cells. Bars indicate mean ± SD

Next, we sought to investigate the effect of ALK-inhibition in combination with high linear energy transfer (LET) particle therapy using carbon ions. Clonogenic survival of all three NSCLC lines was determined at 0 and 4 nM TAE684 and 0, 1, 2 and 4 Gy irradiation with carbon ions. TAE684 significantly sensitized ALK-positive H3122 to carbon irradiation with SER: 1.61 (p < 0.05 at SF 50%) (Fig. 4a). A moderate enhancement of carbon ions was observed in LLC with SER: 1.27 (p < 0.05 at SF50%) (Fig. 4b). Addition of TAE684 does not sensitize A549 cells (SER: 1.03, *p* > 0.05, at SF50%) (Fig. 4c). Together these data support beneficial effects of combined carbon ion therapy and TAE684 in ALK-positive NSCLC.

## Discussion

**Fig. 5:**
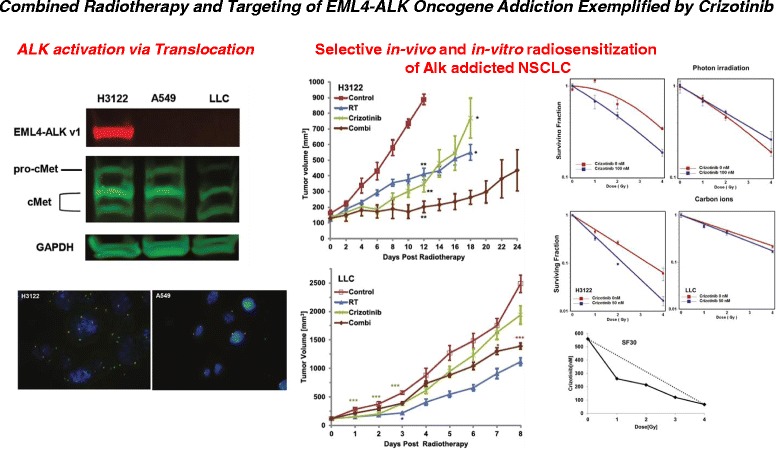
Selective radiosensitization of EML4-ALK oncogene addicted tumors exemplified by crizotinib. A schematic overview of another study conducted in frame of the KFO-214 evaluating the impact of the first generation ALK inhibitor crizotinib on tumor response to radiotherapy [30]. Different tumor cell lines were profiled for their addiction to ALK signaling by western (ALK activation) and FISH (ALK translocation). Crizotinib was originally intended to target cMET, hence cMET signaling was also investigated in all evaluated cell lines. Comprehensive in-vivo tumor growth delay studies revealed selective radiosensitzation of ALK addicted NSCLC cell lines. This was in line with synergistic effects of dual combination observed after photon orcarbon irradiation, respectively, by clonogenic survival assay and isobologram analysis. Together with data reported on second-generation ALK inhibitor TAE684 here, our data support the combination of this class of agents with radiotherapy in ALK addicted NSCLC

Here we report on beneficial effects of combined ALK-inhibition and radiotherapy in NSCLC with EML4-ALK-fusion leading to a constitutive activation of ALK-signaling. Inhibition of ALK-signaling by TAE684 potently and selectively augmented antiproliferative and pro-apoptotic effects of irradiation in ALK-positive tumors. This combination further elicited synergistic effects in reducing the clonogenic survival of ALK-positive H3122 cells. In contrast to ALK-positive H3122 tumor cells, addition of TAE684 does not affect the antiproliferative effect of radiotherapy in two other NSCLC lacking ALK-activation. While the clonogenic survival was moderately reduced by addition of TAE684 to radiotherapy in A549 cells, TAE684 exerted a radioprotective effect in LLC cells as shown by clonogenic survival assays. This data suggests a narrowed indication for combined radiotherapy and ALK-inhibition only in NSCL tumors with aberrant ALK-activation. In contrast to conventional x-ray irradiation, TAE684 moderately sensitized LLC cells (SER: 1.27) when combined with carbon ion irradiation. Hence, radiation quality specific differences may exist in NSCLC response to ALK-inhibitors that warrants further investigation. This is in line with recently reported data on differential phosphoproteome response of NSCLC cells to conventional radiotherapy vs. proton and carbon irradiation [29]. Together with data of enhanced eradication of otherwise radioresistant tumor stem cells by carbon ions, these data indicate further exploration of distinct radiobiological features of different radiation qualities [30]. The potent sensitizing effects of TAE684 on carbon ions observed in ALK-positive H3122 cells (SER: 1.61) suggest further evaluation of this combination in the emerging field of high-LET particle therapy. These data are in complete alignment we recently reported data for combined crizotinib and radiotherapy in NSCLC based on tumor ALK status as schematically summarized in Fig. 5 [30].

High-precision irradiation with carbon ions has shown promising clinical outcomes in NSCLC [31, 32]. Addition of ALK-inhibitors in ALK-positive NSCLC may therefore augment local control effects of radiotherapy and in parallel provide a systemic therapy option to prevent distant tumor growth as the key pattern of therapy failure. Promising data on beneficial effects of combined ALK-inhibition and carbon ion irradiation reported in this study suggest further validation of this concept in preclinical in-vivo tumor models.

TAE684 is a potent small-molecule inhibitor of ALK activity that was shown to block the growth of ALK-dependent cells [25]. In contrast to the FDA/EMA approved, less specific ALK-inhibitor Crizotinib, the selectivity of TAE684 for ALK inhibition was demonstrated across a panel of 22 Kinases and between 100 to 1000 fold higher concentrations of TAE684 was required to inhibit other tyrosine kinases [25]. It was initially identified to inhibit proliferation of ALCLs that harbor the t (2; 5) (p23; q35) chromosomal translocation between ALK and NPM. NPM-ALK is a potent oncogene, possessing strong transforming ability in a wide array of different cell types in vitro and hematopoietic cell lines such as the myeloid line 32Dcl3 [33]. The 80-kD fusion protein is an oncogenic tyrosine kinase that activates downstream signaling pathways related to mitogenic, antiapoptotic and possibly DNA repair capabilities such as PI3K-Akt and JAK-STAT pathways [34–38]. Further studies are needed to identify the relevance of different proposed ALK-downstream signaling pathways in the radiosensitizing effects of TAE684 reported here.

NSCLC is one of the most challenging malignancies, although its prognosis has improved due to new therapeutic agents and better combined therapy regimens. The introduction of FDG-PET-CT has reduced the number of falsely classified locally advanced patients and together with improved target volume delineation and radiotherapy techniques contributed to a better patient outcome. Despite all these progresses, long-term survival is still limited in patients presenting at locally advanced or metastatic stages. Thus, personalized therapies based on individual tumor characteristics are urgently needed for NSCLC patients. The development of ALK inhibitors has provided a promising therapeutic opportunity for treatment of advanced metastatic NSCLC [39, 40]. Based on our data, a relatively large fraction of patients with locally advanced disease and ALK-positive tumors may benefit from combined ALK inhibition and radiotherapy. Hence, in addition to the current practice in stage IV disease, routine examination of ALK status might be warranted in locally advanced Stage III NSCLC prior to radiotherapy. In analogy to combined EGFR-inhibition (cetuximab) and radiotherapy in HNSCC, it is conceivable that ALK-inhibition may provide a less toxic alternative to chemotherapy for concurrent treatment of ALK-positive NSCLC with radiotherapy [41]. Our data unequivocally indicate further in-vivo and clinical exploration of this favorable combination in ALK-fusion-harboring NSCLC.

## Conclusions

In this report, we demonstrated that concurrent inhibition of ALK in combination with irradiation reduced the proliferative capacity and enhanced apoptosis selectively in human H3122 NSCLC tumors with EML4-ALK translocation. Based on the clonogenic survival data, a synergistic activity was further observed between irradiation and TAE684 treatment in H3122 cells. ALK-inhibition further sensitized NSCLC cells to particle therapy with carbon ions. To our knowledge, this is the first demonstration of potent radiosensitizing effects of TAE684 in NSCLC cells harboring EML4-ALK fusion gene. This study constitutes a critical step towards clinical translation of this favorable combination in NSCLC.

## Abbreviations

ALCL: Anaplastic large cell lymphoma
ALK: Anaplastic lymphoma kinase
CT: Computed tomography
DMEM: Dulbecco’s modified Eagle’s Medium
EGFR: Epithermal growth factor receptor
EML4: Echinoderm microtubule-associated protein-like 4
FBS: Fetal bovine serum
FDG-PET-CT: Fluorodeoxyglucose- positron emission tomography-CT
LET: Linear energy transfer
LLC: Murine Lewis Lung Cancer
NPM: Nucleophosmin
NSCLC: Non-small cell lung cancer
PI3K: Phosphoinositide 3-kinase
RTKi: Receptor tyrosine kinase inhibitor
SD: Standard deviation
SER: Sensitizer enhancement ratio
SF: Survival fraction
TKI: Tyrosine kinase inhibitors
